# Dermoscopic Features Summarization and Comparison of Four Types of Cutaneous Vascular Anomalies

**DOI:** 10.3389/fmed.2021.692060

**Published:** 2021-06-28

**Authors:** Jing Gao, Wenmin Fei, Changbing Shen, Xue Shen, Minghui Sun, Ning Xu, Qing Li, Cong Huang, Tingfang Zhang, Randy Ko, Yong Cui, Chunjun Yang

**Affiliations:** ^1^Department of Dermatology, The Second Affiliated Hospital, Anhui Medical University, Hefei, China; ^2^Department of Dermatology, China-Japan Friendship Hospital, Beijing, China; ^3^Institute of Skin Health, China-Japan Friendship Hospital, Beijing, China; ^4^Graduate School, Peking Union Medical College and Chinese Academy of Medical Sciences, Beijing, China; ^5^Department of Dermatology, Peking University Shenzhen Hospital, Shenzhen, China; ^6^Shenzhen Key Laboratory for Translational Medicine of Dermatology, Shenzhen Peking University–The Hong Kong University of Science and Technology Medical Center, Shenzhen, China; ^7^Department of Dermatology, Chengdu Second People's Hospital, Chengdu, China; ^8^Division of Molecular Medicine, Department of Internal Medicine, University of New Mexico Health Sciences Center, Albuquerque, NM, United States

**Keywords:** dermoscopy, features, cutaneous vascular anomalies, summarization, comparison

## Abstract

**Objective:** Dermoscopic features of cutaneous vascular anomalies have been reported, but the described features currently known are limited and not well-understood. The aim of this study is to comprehensively summarize and compare the dermoscopic features of the four different types of cutaneous vascular anomalies [infantile hemangiomas (IH), cherry angioma (CA), angiokeratomas (AK), and pyogenic granuloma (PG)] in the Chinese Han population.

**Materials and Methods:** Dermoscopic features of 31 IH, 172 CA, 31 AK, and 45 PG were collected based on the contact non-polarized mode of dermoscopy at 20-fold magnification. Dermoscopic features including background, lacunae, vessel morphology and distribution were collected and summarized. Additionally, we compared these features by age stage, gender, and anatomical locations in CA.

**Results:** The dermoscopic features of IH included the red lacunae, red/red-blue/red-white backgrounds, and vessel morphology such as linear curved vessels, serpiginous vessels, coiled vessels. For CA, the lacunae appeared reddish brown to reddish blue or only red. In terms of vascular morphology, serpentine vessels, coiled vessels, looped vessels, and curved vessels could be seen in the lesions. A few lesions were black or presented with a superficial white veil. There were statistical differences in red background (*P* = 0.021), unspecific vessel distribution (*P* = 0.030), black area (*P* = 0.029), and white surface (*P* = 0.042) among different age groups. Red-brown lacunae (*P* = 0.039), red-blue (*P* = 0.013), red-white background (*P* = 0.015), black area (*P* = 0.016), and white surface (*P* = 0.046) were of statistical difference in terms of the locations of lesions. Lacunae were also observed in AK, which presented with red, dark purple, dark blue, black. Global dermoscopic patterns that were characterized by a homogeneous area were obvious in all PG lesions, among which 30 (66.7%) were red-white and 15 (33.3%) were red. As for local features, “white rail” lines were detected in 19 (42.2%) lesions and white collarette was seen in 34 (75.6%) lesions.

**Conclusions:** Dermoscopy is an applicable diagnostic tool for the diagnosis of cutaneous vascular anomalies. It is necessary to take into account the age stage and lesion location when we diagnose CA using dermoscopy.

## Introduction

Vascular anomalies account for a considerable number of patients in the dermatology and surgical outpatient departments, which can be grouped into vascular tumors and vascular malformations. Some vascular tumors are proliferative and have the potential to fade away spontaneously, while vascular malformations normally stay stagnant over a long period of time (1). Timely and accurate diagnosis of vascular anomalies are essential for treatment and management, but remains a challenge for clinicians (2). Infantile hemangioma (IH), pyogenic granuloma (PG), angiokeratoma (AK), and cherry angiomas (CA) are common cutaneous vascular anomalies. According to the latest classification for vascular anomalies by the International Society for Vascular Anomalies (ISSVA), IH and PG are classified into the benign vascular tumors, angiokeratoma (AK) is classified into provisionally unclassified vascular anomalies (3). However, CA are not included in the current classification by the latest ISSVA (4).

IH is one of the most common cutaneous vascular anomalies present in infants, with the affected population being approximately 2–4.5% (5). The majority of IHs can be identified by clinical features, but some cases may still be misdiagnosed. CA, also known as cherry hemangiomas, adult hemangiomas, or senile angiomas as their number tends to increase with age, are cherry-red papules on the skin containing abnormal growth of blood vessels. CA are the most common vascular anomalies in adults, and elderly patients are especially concerned about the possible progression to malignancy. AK is a rare vascular malformation of the upper dermis that tends to have a distinct clinical presentation without self-limiting behavior. Clinically, the presentation of AK is often a dark nodule or macule which may be easily confused with malignant tumors, such as malignant melanomas, or pigmented basal cell carcinomas. A previous study reported that 20% of AK lesions were clinically diagnosed as melanomas (6). PG is a common and benign vascular tumor that can be diagnosed correctly by medical history and appearance. However, a survey indicated that 38% of the clinical diagnosis of PG proved to be inaccurate (7).

Dermoscopy has been widely accepted as a useful non-invasive diagnostic tool for various pigmented and non-pigmented skin disorders for increasing the diagnostic accuracy and avoiding unnecessary biopsies (8–12). The dermoscopic features of the above four types of cutaneous vascular anomalies have been previously reported in a few cases (6, 7, 12–17). However, dermoscopic features described in these studies are limited and not well-understood. Hence, this study aimed to comprehensively summarize the dermoscopic features of the four types of cutaneous vascular anomalies in the Chinese Han population.

## Materials and Methods

### Study Design

This study was a morphological study carried out at the Second Affiliated Hospital of Anhui Medical University and China-Japan Friendship Hospital from June 1st, 2017 to October 31st, 2020. Approval was granted by the two institutions' ethics committees and conformed to the Declaration of Helsinki. All patients or their guardians provided informed consents. All lesions were clinically diagnosed by two associate chief physicians or chief physicians. If there was a disagreement between the two experts, the lesion would be discussed by an additional chief physician. The exclusion criteria included the use of: topical beta blocker, or intra-lesion injection, or laser treatment in the past 2 weeks, or a history of hematological malignancy. One lesion of each patient was selected to undergo dermoscopic examination.

### Imaging Procedure

Dermoscopic examinations were performed with a digital dermoscopy system (Medicam 800HD, FotoFinder Systems GmbH, Birbach, Germany) at a 20-fold magnification. The contact non-polarized mode of dermoscopy was utilized. In order to acquire better visualization, minimal pressure was applied and ultrasound gel was used to preserve vessels' morphology. Dermoscopic images were evaluated by two independent dermatologists (JG and WF). Any poor-quality images were excluded. We developed a list of features based from literature review and preliminary observation which included lacunae, background, vessel morphology and distribution. Two dermatologists (WF and XS) completed the list independently and also supplemented new items features beyond the list if necessary. If there was a disagreement between the two experts or any supplementary information, a consensus meeting with other experts was held to settle any discrepancies. The dermoscopic features described in this study refer to the standardized dermoscopic terminology from an expert consensus on behalf of the International Dermoscopy Society (18, 19).

### Divisions of Body Locations and Age Stages

Previous studies showed that there were differences of dermoscopic features and patterns in different age stages, gender, body location of lesions (20, 21). General information such as age, gender, anatomical locations were collected in this study. The body locations were divided into four groups, including upper limbs, lower limbs, head and neck, and trunk. The hips were classified into the lower limbs category, and the perineum was categorized into the lower trunk. Gender, age, and location difference were analyzed in CA. For the CA patients, we divided the patients' age into three stages: <18 years old group (children), 18–60 years old group (adults), and >60 years old group (elders).

### Statistical Analysis

The continuous data are represented as mean (M) ± standard deviation (SD), and the categorical data are shown as number (N) and percentage (%). The categorical variables were compared by utilizing the Chi-square test. Regarding the Chi-square test in R x C contingency tables, if the expected count (T) of any grid in the R x C contingency tables were <1, or the number of grids with 1 ≤ T < 5 exceed 20% of the total number of grids, then Fisher's exact test was used to calculate the *P*-value. Statistical package for social science (SPSS), version 22.0 (IBM Corp., Armonk, NY, USA) was used for all analysis. A two-sided *P*-value < 0.05 was considered statistically significant in all analysis.

## Results

**Figure 1:**
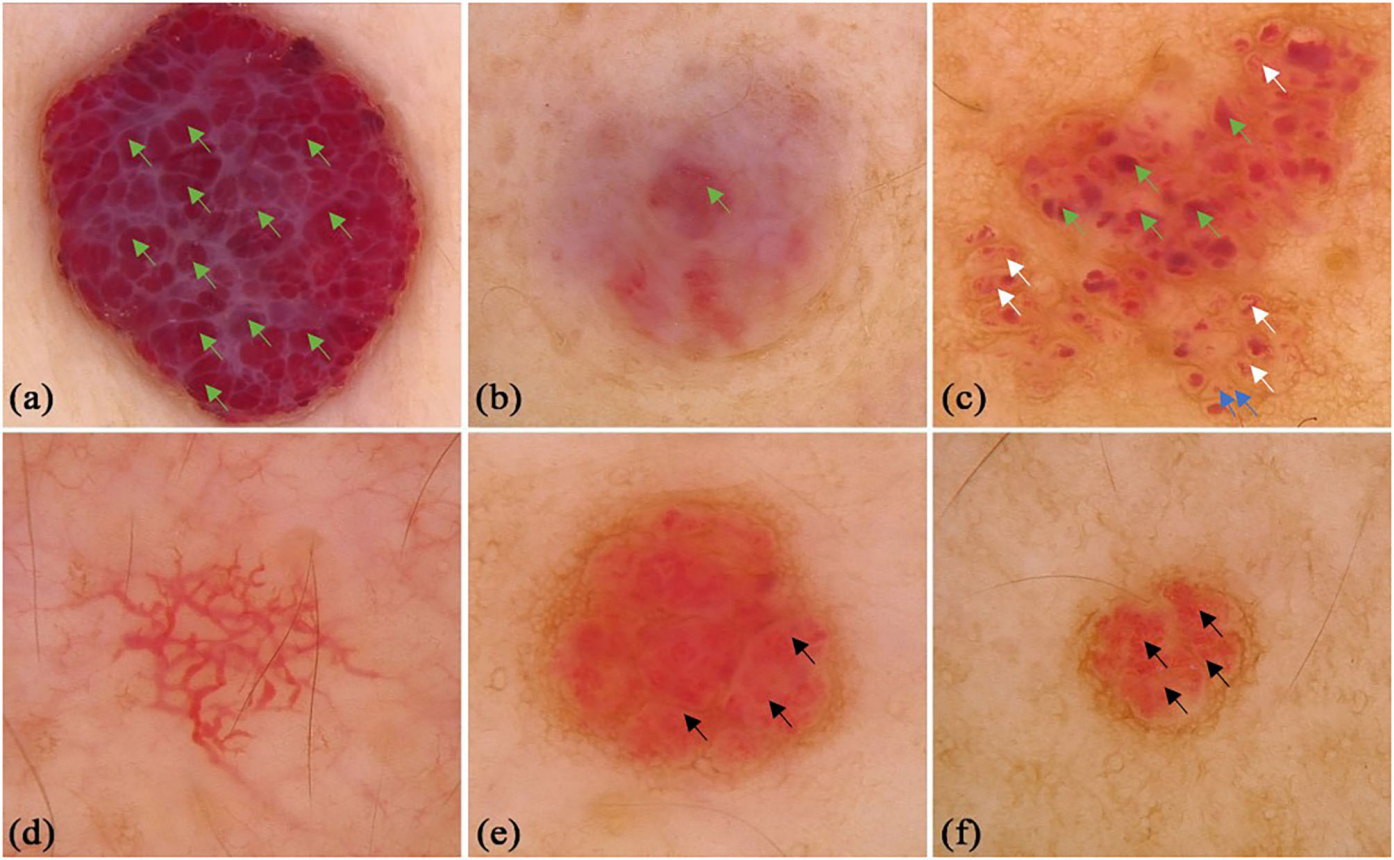
Dermoscopic features of infant hemangiomas. **(a)** red-blue background, clustered red lacunae (green arrows); **(b)** red-white background, red lacunae (green arrows); **(c)** red background, red lacunae (green arrows), red globules (blue arrows) and helical vessels (white arrows); **(d)** reticular vessel distribution; **(e)** clustered serpentine vessels (black arrows); **(f)** serpentine vessels (black arrows).

There were 31 patients with IH, including 8 male and 23 female infants. The lesions were located on the upper limbs (*n* = 2, 6.5%), lower limbs (*n* = 4, 12.9%), head and neck (*n* = 12, 38.7%), and trunk (*n* = 13, 41.9%). Red lacunae was seen in 10 (32.3%) lesions. Red, red-blue, and red-white backgrounds were seen in 19 (61.3%), 1 (3.2%), and 4 (12.9%) lesions, respectively. There were definitive vascular structures besides lacunae in some lesions. Linear curved vessels could be discovered in 2 (6.5%) lesions, serpiginous vessels in 9 (29.0%) lesions, and coiled vessels in 6 (19.4%) lesions. In the lesions that contained multiple structures, reticular vessel distribution was seen in 9 (29.0%) lesions, 12 (38.7%) lesions with unspecific vessel distribution, while 9 (29.0%) lesions with clustered vessel distribution. Some of the classical and specifical dermoscopic features were showed in Figure 1.

**Figure 2:**
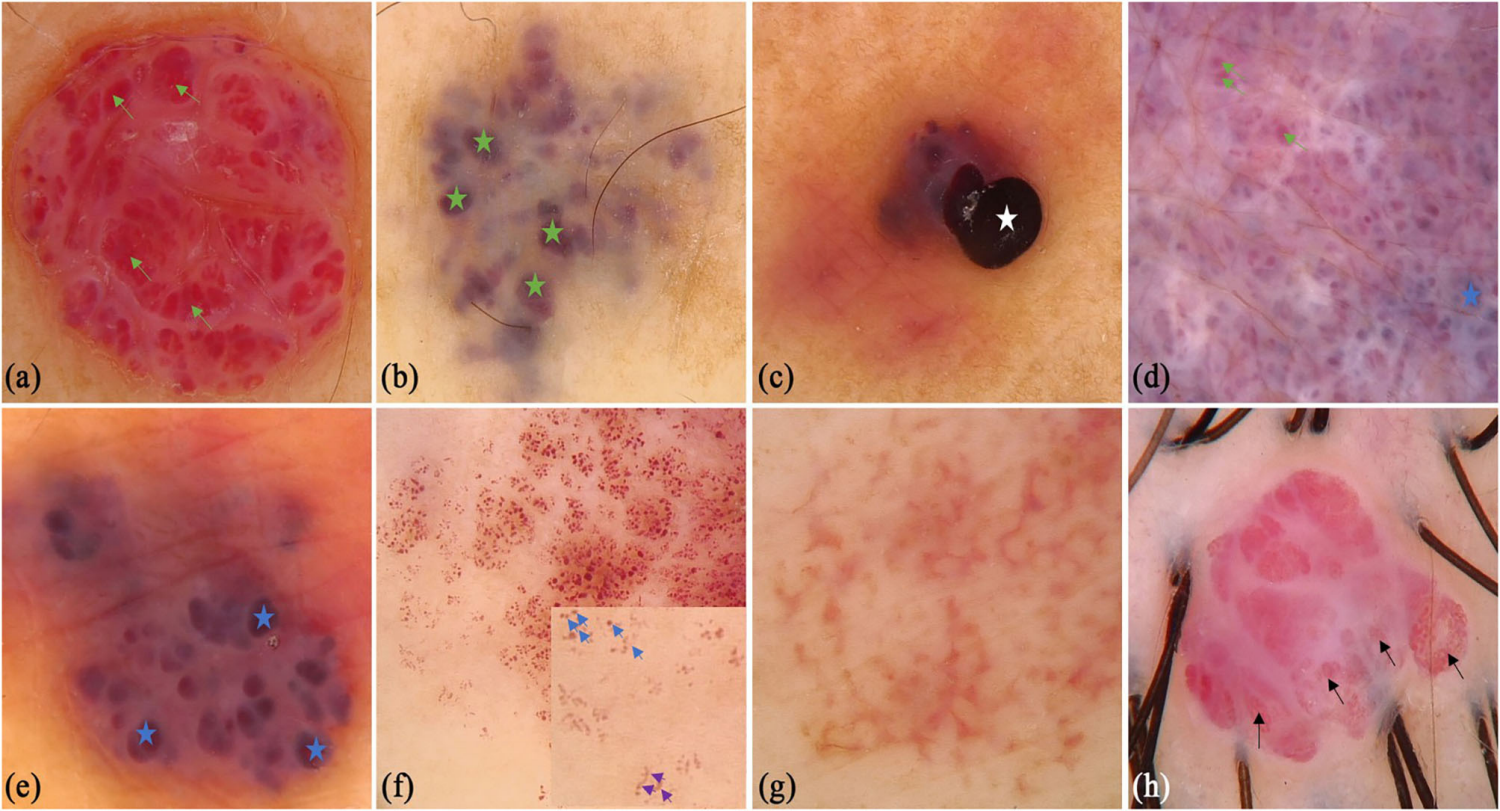
Dermoscopic features of cherry angioma. **(a)** Typical dermoscopic appearance: red background, clustered red lacunae (green arrows), white surface; **(b)** red-white background, red-brown lacunae (green stars); **(c)** red background and black area (white star); **(d)** red-blue background, clustered red (green arrows) and red-blue lacunae (blue stars), white surface; **(e)** red-blue background, red-blue lacunae (blue stars), white surface; **(f)** red background, red globules (blue arrows) and curved vessels (purple arrows), clearer in the small box; **(g)** reticular vessel distribution; **(h)** clustered serpentine vessels (black arrows).

A total of 172 CA patients (92 males and 80 females, mean age was 39.38 ± 18.41 years old) were included in the study. There were 41 (23.8%) lesions located on the upper limbs, 28 (16.3%) lesions on the lower limbs, 52 (30.2%) lesions on the head and neck, and 51 (29.7%) lesions on the trunk. Typical lacunae existed in 127 (73.84%) lesions, among which red lacunae were seen in 78 (45.3%) lesions, red-brown lacunae in 24 (14.0%) lesions, and red-blue lacunae in 42 (24.4%) lesions. Different colored backgrounds were identified in 158 (91.86%) lesions. Among the lesions, 77 (44.8%) were red, 52 (30.2%) were red-blue, and 34 (19.8%) were red-white. Regarding the vessel morphology, linear curved vessels were found in 29 (16.9%) lesions, serpiginous vessels in 27 (15.7%) lesions, coiled vessels in 33 (19.2%) lesions, looped vessels in 7 (4.1%) lesions, and dotted vessels in 14 (8.1%) lesions. For the vessel distribution, reticular distribution in 12 (7.0%) lesions, 58 (33.7%) lesions with clustered vessel distribution, and 70 (40.7%) lesions with unspecific vessel distribution. A black area was seen in 24 (14.0%) lesions, and superficial white veil was seen in 63 (36.6%) lesions. Other infrequent vessel morphology in CA, included linear (*n* = 12, 6.98%), looped (*n* = 6, 3.49%), helical (*n* = 6, 3.49%), coiled (*n* = 7, 4.07%), serpiginous (*n* = 1, 0.58%), and branched (*n* = 1, 0.58%) vessels. Some classical and specifical dermoscopic features are shown in Figure 2.

We conducted comparisons of the dermoscopic features of CA (Table 1). There was no difference of dermoscopic features were observed between males and females. There were statistical differences in the red background (*P* = 0.021), clustered vessel distribution (*P* = 0.03), black area (*P* = 0.029), and white surface features (*P* = 0.042) among different age groups. Red-brown lacunae (*P* = 0.039), red-blue (*P* = 0.013), red-white background (*P* = 0.015), black area (*P* = 0.016), and white surface (*P* = 0.046) were of statistical difference in terms of the body locations of the lesions. Red lacunae were most common in head and neck, red background was most common in lower limbs, while a black area was most common in trunk.

**Table 1:** Summarization and comparison of dermoscopic features of cherry angioma.

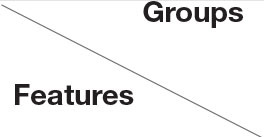	**Sex**			**Age stage**				**Anatomical location**				
	**Male** **(*****n*** **=** **92)**	**Female** **(*****n*** **=** **80)**	***P***	**<18 years** **old (*****n*** **=** **26)**	**18–60 years old (*****n*** **=** **120)**	**>60 years old (*****n*** **=** **26)**	***P***	**Upper limbs (*****n*** **=** **41)**	**Lower limbs (*****n*** **=** **28)**	**Head and neck (*****n*** **=** **52)**	**Trunk** **(*****n*** **=** **51)**	***P***
**Lacunae**												
Red	48 (52.2)	30 (37.5)	0.054	11 (42.3)	55 (45.8)	12 (46.2)	0.944	17 (41.5)	8 (28.6)	28 (53.8)	25 (49.0)	0.156
Red-brown	13 (14.1)	11 (13.8)	0.943	6 (23.1)	14 (11.7)	4 (15.4)	0.303	6 (14.6)	9 (32.1)	3 (5.8)	6 (11.8)	0.013
Red-blue	24 (26.1)	18 (22.5)	0.585	4 (15.4)	30 (25.0)	8 (30.8)	0.419	4 (9.8)	8 (28.6)	12 (23.1)	18 (35.3)	0.039
**Background**												
Red	41 (44.6)	36 (45.0)	0.954	7 (26.9)	62 (51.7)	8 (30.8)	0.021	21 (51.2)	13 (46.4)	20 (38.5)	23 (45.1)	0.668
Red-blue	26 (28.3)	26 (32.5)	0.546	10 (38.5)	31 (25.8)	11 (42.3)	0.155	11 (26.8)	14 (50.0)	12 (23.1)	15 (29.4)	0.082
Red-white	22 (23.9)	12 (15.0)	0.143	3 (11.5)	22 (18.3)	9 (34.6)	0.087	5 (12.2)	1 (3.6)	16 (30.8)	12 (23.5)	0.015
**Vessel morphology**												
Linear curved	18 (19.6)	11 (13.8)	0.310	5 (19.2)	21 (17.5)	3 (11.5)	0.739	9 (22.0)	1 (3.6)	10 (19.2)	9 (17.6)	0.211
Serpiginous	14 (15.2)	13 (16.3)	0.853	2 (7.7)	22 (18.3)	3 (11.5)	0.395	8 (19.5)	2 (7.1)	6 (11.5)	11 (21.6)	0.261
Globules	19 (20.7)	14 (17.5)	0.601	3 (11.5)	28 (23.3)	2 (7.7)	0.140	9 (22.0)	5 (17.9)	8 (15.4)	11 (21.6)	0.824
Looped	4 (4.3)	3 (3.8)	>0.99^*^	0 (0.0)	6 (5.0)	1 (3.8)	0.835	3 (7.3)	0 (0.0)	2 (3.8)	2 (3.9)	0.583^*^
Dots	9 (9.8)	5 (6.3)	0.398	1 (3.8)	9 (7.5)	4 (15.4)	0.347	7 (17.1)	2 (7.1)	2 (3.8)	3 (5.9)	0.135^*^
**Vessel distribution**												
Reticular	4 (4.3)	8 (10.0)	0.147	4 (15.4)	5 (4.2)	3 (11.5)	0.058	4 (9.8)	3 (10.7)	2 (3.8)	3 (5.9)	0.567^*^
Clustered	33 (35.9)	25 (31.3)	0.523	4 (15.4)	41 (34.2)	13 (50.0)	0.030	10 (24.4)	8 (28.6)	16 (30.8)	24 (47.1)	0.103
Unspecific	36 (39.1)	34 (42.5)	0.654	11 (42.3)	49 (40.8)	10 (38.5)	0.959	23 (56.1)	7 (25.0)	21 (40.4)	19 (37.3)	0.068
**Black area**	16 (17.4)	8 (10.0)	0.163	6 (23.1)	18 (15.0)	0 (0.0)	0.029	4 (9.8)	7 (25.0)	2 (3.8)	11 (21.6)	0.016
**White surface**	34 (37.0)	29 (36.3)	0.924	7 (26.9)	41 (34.2)	15 (57.7)	0.042	9 (22.0)	8 (28.6)	25 (48.1)	21 (41.2)	0.046

* *Fisher's exact test was used to calculate the P-values*.

**Figure 3:**
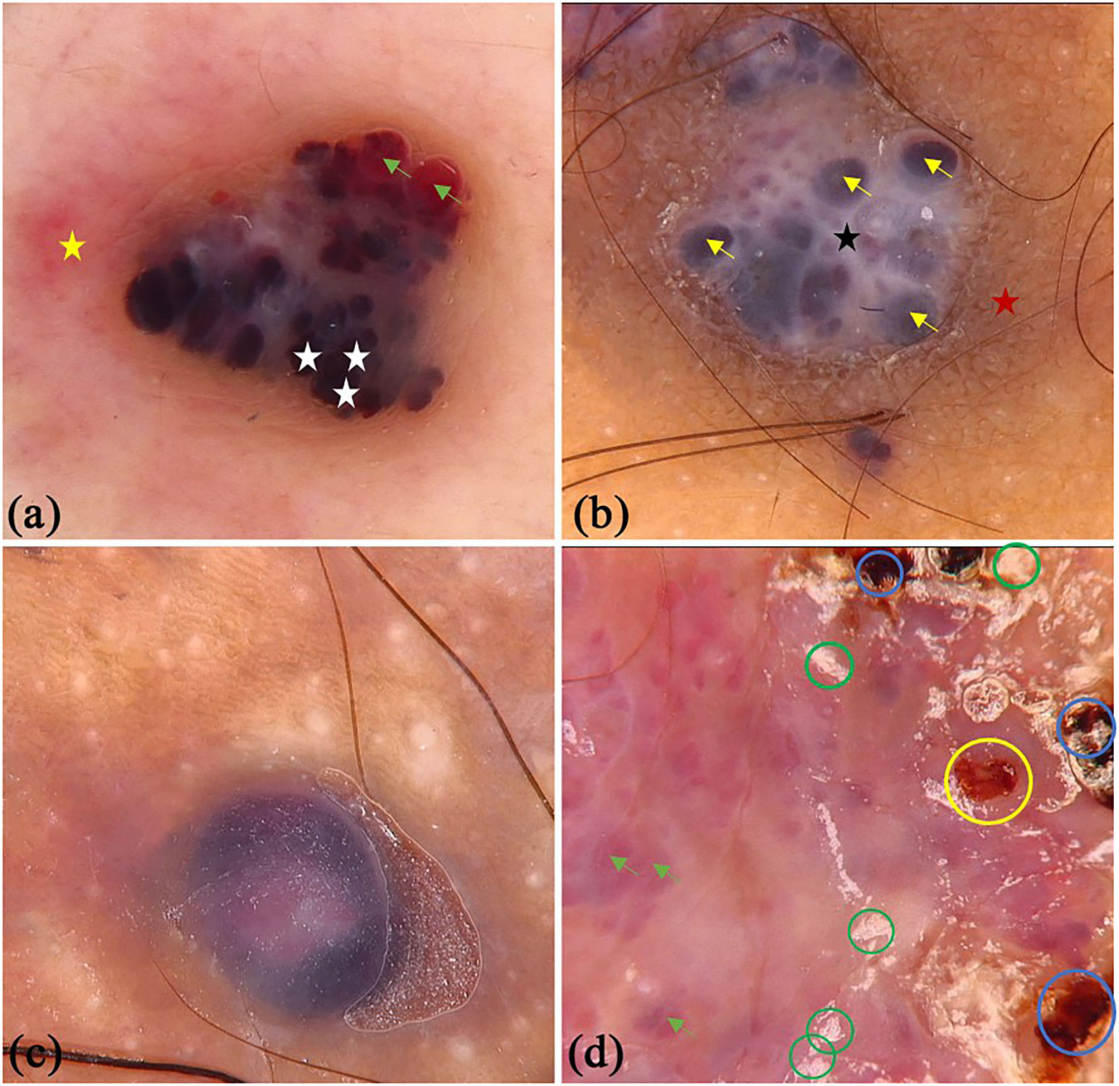
Dermoscopic features of angiokeratoma. **(a)** clustered black area (white star) and red lacunae (green arrows), light white curtain, peripheral redness (yellow star); **(b)** dark purple lacunae (yellow arrows), light white curtain (black star), peripheral pigment network (red star); **(c)** dark blue lacuna; **(d)** red lacunae (green arrows), white scales (green circles), dark red scabs (blue circles) and bleeding (yellow circle).

We also collected 31 patients (16 males and 15 females) with AK, whereas the mean age was 33.29 (SD = 18.03) years old. Some of the classical and specifical dermoscopic features are shown in Figure 3. There were 5 (16.1%), 14 (45.2%), and 12 (38.7%) lesions that were located on the upper limbs, lower limbs, head and neck, respectively. Red lacunae could be seen in 13 (41.9%) lesions, dark purple lacunae in 25 (80.6%) lesions, dark blue in 3 (9.7%) lesions, and black lacunae in 4 (12.9%) lesions. A superficial white veil feature was found in 24 (77.4%) lesions, bleeding in 6 (19.4%) lesions, hemorrhagic crusts in 4 (12.9%) lesions, and scales in 7 (22.6%) lesions. Peripheral redness was seen in 7 (22.6%) lesions and peripheral pigment network in 2 (6.5%) lesions.

**Figure 4:**
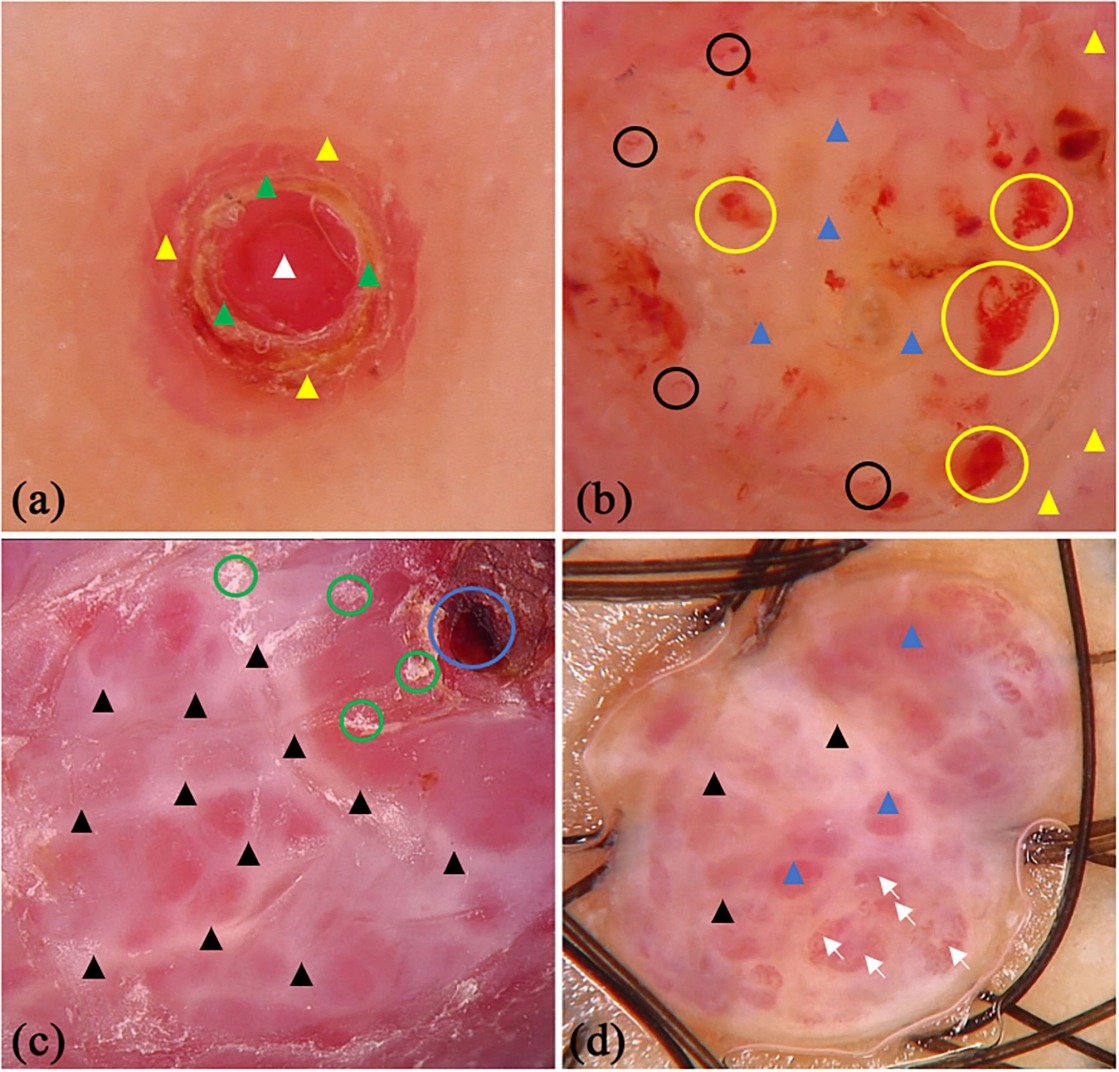
Dermoscopic features of pyogenic granuloma. **(a)** red homogeneous area (white triangle), yellow scabs (green triangles), collar pattern (yellow triangles); **(b)** red-white homogeneous area (blue triangles), white collar pattern (yellow triangles), vascular structures (black circles) and bleeding (yellow circles); **(c)** “white rail” lines (black triangles), dark red scabs (blue circles) and white scales (green circles); **(d)** red-white homogeneous area (blue triangles) and “white rail” lines (black triangles), serpentine vessels (white arrows).

There were 45 patients (29 males and 16 females) with PG in this study, whereas the mean age was 44.11 (SD = 16.47) years old. Some of the classical and specifical dermoscopic features are shown in Figure 4. There were 26 (64.4%) lesions located on the upper limbs, 3 (6.7%) lesions on lower limbs, 15 (33.3%) lesions on head and neck, and only 1 (2.2%) lesion located on trunk. Global dermoscopic patterns that were characterized by a homogeneous area were obvious in all lesions, among which 30 (66.7%) were red-white and 15 (33.3%) were red. As for local features, “white rail” lines were detected in 19 (42.2%) lesions and white collarette in 34 (75.6%) lesions. Furthermore, vascular structures were observed in 19 (42.2%) lesions. Other atypical dermoscopic features included superficial ulcer (*n* = 2, 4.4%), thick crust (*n* = 10, 22.2%), bleeding (*n* = 4, 8.9%), scale (*n* = 13, 28.9%), and a central white veil (*n* = 2, 4.4%).

## Discussion

Early and accurate diagnosis represents the safest strategy in the treatment of cutaneous vascular anomalies. This may reduce disease-related mortality, avoid unnecessary surgery, relieve the suffering of patients and ease potential financial burden. However, it can be difficult to manage as a result of the atypical clinical presentation and the progression being similar to malignancy. Dermoscopy is a non-invasive tool that has been applied clinically to support the diagnosis and differential diagnosis of skin tumors (22–25), here we present the dermoscopic differential diagnosis of four types of cutaneous vascular anomalies from other skin disorders (25–31) (Table 2). With dermoscopy, predominant vascular structures and other recognizable concomitant features can be observed. This may lead to improved accurate diagnosis of cutaneous vascular anomalies that originate from cutaneous vessels, if promptly recognized. This study describes the dermoscopic features of four types of cutaneous vascular anomalies in 279 patients.

**Table 2:** Dermoscopic differential diagnosis of four types of cutaneous vascular anomalies from other skin disorders.

**Category**	**Diseases**	**Common dermoscopic features**
Four types of cutaneous vascular anomalies	IH	Red/red-blue/red-white background, clustered red lacunae; polymorphous vascular structures: red globular and helical vessels; clustered serpentine vessels (25).
	CA	Red/red-white/red-blue background, red/red-blue/dark lacunae, white surface; red globular and curved vessels, clustered serpentine, blue-whitish veil, ulceration, rainbow pattern.
	AK	Clustered black to dark red or purple-blue lacunae and whitish veil, peripheral redness; peripheral pigment network; white scales, dark red scabs and bleeding, blue whitish veil, ulceration, rainbow pattern (16).
	PG	Reddish homogeneous area, white collarette, “white rail” lines that intersect the lesion and ulceration (15). Red or white homogeneous area, surrounded by a white collar-like structure, thick tortuous blood vessels can be seen in the skin lesions. reddish homogeneous areas associated with “white rail” lines (17).
Other differential skin disorders	Foreign body granuloma	Orange background, white or blue-gray homogeneous areas; branched vessels (12).
	Verruca vulgaris	Multiple tightly packed papillae with red, brown dots, linear or looped vessels; white halos around looped vessels in some cases; irregular red, black, brown bleeding or scabs (12, 29, 30).
	Malignant melanoma	Negative or atypical pigment network; atypical streaks (radial streaming or pseudopods); blue-whitish veil; irregular dots or globules; irregular blotch; polychromatic; shiny white streaks; vascular pattern: pink areas, irregular dots or looped vessels (12).
	Spitz nevus	Starburst pattern, pink or pigment homogeneous pattern, globular pattern, atypical pattern, reticular pattern (12, 31).
	Bowenoid papulosis	Scattered dot, curved, looped, or coiled vessels; blue-gray or gray-brown homogeneous area (12, 27).
	Kaposi sarcoma	Bluish-reddish coloration including purple; multicolored areas showing various colors of the rainbow spectrum; scaly surface; small brown globule or blotch; reticular coarse vessels; shiny white streaks (12, 28).
	Dermatofi-brosarcoma protuberan	Topographic pattern background (pink, buff or tan); branched vessels (clustered or disseminated); shiny white streaks; delicate pigment network; structureless area or hypopigmented area (12).
	Proliferating trichilemmal tumor	Pink background, shiny white streaks, irregular and crown vessels, (sometimes) with hairs and ulceration (12, 26).

Our study indicated that nearly three quarters of the lesions showed lacunae, which is regarded as a diagnostic clue for IH and CA (32). Lacunae corresponded to dilated vessels that are filled with red blood cells in the dermal papillae. Although lacunae appear in a variety of colors, their overall hue is red. The overall hue of backgrounds in IH and CA were red as well and the observed three colors in our study were consistent with a previous study (17). Besides lacunae, there are other vessels structures in IH and CA. The arrangement of vessels includes reticular, clustered, and unspecific vessel distributions, and the last one is the most common. It is worth noting that some lesions contain only one structure, and the possibilities of IH or CA should be taken into consideration when this is observed. In the vascular structures that have been reported before, we found that coiled vessel features were the most common. The appearance of a well-defined black area corresponds to thrombogenesis under histopathology (33). The superficial white veil feature, which were detected frequently, histopathologically resulted from the presence of an acanthotic epidermis with compact hyperkeratosis (34).

Typical dermoscopic features of AK include a well-demarcated, round lacunae and a whitish veil, whose histopathological basis are consistent with their equivalents in IH and CA (35). The overall hue of lacunae in AK is dark, with dark purple being the most common one in our study. Dark blue, purple, or a black color corresponds to vascular spaces that are partially or completely thrombosed. Peripheral redness, which likely represents inflammation of the lesion and erythrocyte extravasation in the papillary dermis, were seen in seven lesions. Two lesions presented a peripheral pigment network due to hyperpigmented rete ridges, which is akin to the network observed in dermatofibromas (36). Hemorrhagic crusts were detected in 12.9% lesions, which is far lower than the 53% reported in previous study (16). Scale features, which have been reported in previous study (6), were discovered in 22.6% lesions (7 of 31). Also, scale is a clinical manifestation of AK.

From our observation, a homogeneous area including reddish and red-whitish features, were the most frequent dermoscopic structure associated with PG, which is consistent with a previous study (7). The histopathological correlation of a homogeneous area may be attributed to the presence of numerous small capillaries or proliferating vessels which served as a foundation for various vascular structures. The white collarette, which was seen in 75.6% of lesions in our study, corresponds to the hyperplastic adnexal epithelium that partially or totally surrounds the lesion at the periphery of PG. This frequency of the white collarette is similar to previous study (7). The “white rail” lines may correspond to the fibrous septa that surround the capillary tufts or lobules (15), but it was not so common as white collarette features. The “white rail” lines can be observed in dermatofibromas and fully regressive melanomas when a polarized mode is used (37, 38). Erosion, bleeding, and scabbing are common clinical features of PG. These often lead to a misdiagnosis as malignant tumors which present as a dark-red or black homogeneous structure, hemorrhagic crusts, or ulceration revealed with dermoscopy. Serous exudation present explains the observation of orange-yellow crusts.

Other than the reported features in previous literatures, we observed some other dermoscopic findings in these cutaneous vascular anomalies. In IH and CA, some other morphology features include irregular linear, looped, helical, coiled, serpiginous, and branched vessels which were detected in our study. These features seem to be associated with dilated and proliferated vessels in the upper dermis and some of them had diagnostic specificity for some malignant tumors. For example, coiled vessels in Bowen's disease (39). In our experience, scales and partial whitish veil features can also be seen in PG, which had never been described previously. These two features could appear in multiple diseases, for instance, scales may be present in inflammatory skin diseases and whitish veil in skin tumors.

We analyzed differences in gender, age stage, and location of the lesions. Red lacunae was more commonly observed in females. They may be linked to the higher levels of estrogen in female patients (40). Red-blue background and lacunae were more common in adults, which may be a signs of thrombi within the vascular spaces (17). This may also explain why black areas were more common in older patients. Additionally, dilated vessels in CA were veins, with the addition of some vessels in deeper dermis which may contribute to the color of red-blue in the adult. Another age-related dermoscopic feature was the presence of a superficial whitish veil, which may be explained as IH is self-limited while CA is not. In relation to anatomical areas, the feature of a black area, red-brown lacunae, and red-white background were the most prevalent in the trunk region.

It should be noted especially that there are some limitations of our study. One is that the diagnosis of four types of cutaneous vascular anomalies were based on clinical and dermoscopic features by two dermatologists and no histopathological examination was performed. The variations in anatomical sites and some newly discovered features have not been well-studied by histological analysis. Another one is the limited study sample size of IH, AK, and PG; many more numbers of these three cutaneous vascular anomalies patients should be recruited for a future study.

## Conclusion

Dermoscopic evaluation is an easy, applicable, and ancillary diagnostic tool for the diagnosis of cutaneous vascular anomalies. In clinical practice, when dermatologists diagnose CA with dermoscopy, it is necessary to consider that dermoscopic features vary in age stages and different anatomical sites.

## Data Availability Statement

The original contributions generated for this study are included in the article, further inquiries can be directed to the corresponding authors.

## Ethics Statement

The studies involving human participants were reviewed and approved by The Second Affiliated Hospital of Anhui Medical University and China-Japan Friendship Hospital. Written informed consent in this study was provided by participants or the participants' legal guardian/next of kin.

## Author Contributions

CY and YC designed this study. WF, XS, JG, CS, MS, NX, QL, and TZ collected the clinical and dermoscopic data. CS, JG, RK, and WF analyzed of data and interpreted the analysis results. JG, WF, CS, and XS prepared the manuscript. RK, CH, CS, CY, and YC revised this manuscript. All authors contributed to the article and approved the submitted version.

## Conflict of Interest

The authors declare that the research was conducted in the absence of any commercial or financial relationships that could be construed as a potential conflict of interest.
